# An alternative surgical approach to subclavian and innominate stenosis: a case series

**DOI:** 10.1186/1749-8090-5-73

**Published:** 2010-09-23

**Authors:** Amina Khalil, Samer AM Nashef

**Affiliations:** 1 Department of Cardiothoracic Surgery, John Radcliffe Hospital, Headley Way, Headington, Oxford OX3 9DU, UK; 2 Papworth Hospital, Cambridge CB23 3RE, UK

## Abstract

We report three cases of symptomatic stenosis of the great vessels or supra-aortic trunks successfully treated surgically with aorto-subclavian and aorto-innominate bypass. Two were performed via manubriotomy and a third case via standard median sternotomy because of concomitant coronary revascularisation. There was complete symptomatic relief on follow-up, and radiological imaging confirmed good flow in the grafts and post-stenotic arteries.

## Background

Like other arteries, the innominate, left common carotid and subclavian arteries or supra-aortic trunks (SATs) can be affected by atherosclerosis. Many patients with SAT disease are asymptomatic, but some may present with symptoms of cerebral or limb ischaemia. The use of endovascular intervention for SAT occlusive disease is increasing but open surgical reconstruction remains an effective treatment option with good long term results. Although the cervical approach for the treatment of SAT disease has proven to be a good surgical option over the years, a transthoracic approach can provide durable results particularly when the disease process affects all three trunks or involves long segments [1]. The morbidity associated with the transthoracic route may be reduced by using a less invasive approach such as manubriotomy. A short summary of clinical presentation, the surgical technique employed and the outcomes forms the basis of the present case series.

### Case 1

A 64-year-old male presented with frequent episodes of dizziness after myocardial infarction. Ambulatory 24-hour cardiac monitoring showed periods of asystole, and a dual chamber pacemaker was implanted. The patient remained symptomatic with the same frequency of dizzy spells and reported syncopal episodes precipitated by left arm exertion. Contrast-enhanced spiral computerised tomography (CT) revealed disease at the origin of all great vessels, with an irregular 50% stenosis at the origin of innominate artery, a 70% stenosis at the origin of the right subclavian and a 30% stenosis of at the origin of left common carotid artery. The first 15-mm segment of the left subclavian artery proximal to the origin of left vertebral artery was totally occluded. The distal left subclavian filled by retrograde flow through the ipsilateral vertebral artery (subclavian steal syndrome).

**Figure 1:**
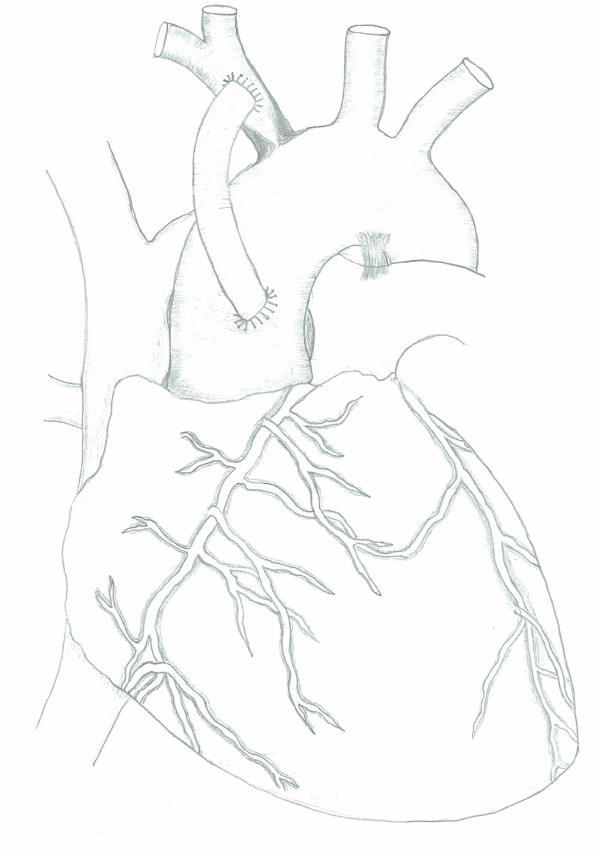
**Aorto-subclavian Bypass Graft**.

At operation, the skin was incised above the clavicle from the left mid-clavicular point to the suprasternal notch and the incision extended vertically downwards towards the manubriosternal junction. This was followed by a vertical manubriotomy extending laterally to the left, stopping short of the internal mammary pedicle. A self-retaining retractor was used to separate the two halves of the manubrium and to elevate the sternal edge on the left side, giving good access to both the ascending aorta and the distal subclavian artery. Under full heparinisation, the artery was clamped 3 cm distal to the occlusion and a polytetrafluoroethylene graft was anastomosed end-to-side beyond the occlusion using 5/0 monofilament polypropylene. The graft was trimmed to size and anastomosed to the ascending aorta using a partial occlusion clamp (Fig 1). Heparin was reversed and the incision closed over a small suction drain with a figure-of-8 single sternal wire and standard soft-tissue closure. The patient made an uneventful recovery. Repeat CT at 2 weeks demonstrated good antegrade filling of the distal left subclavian and vertebral artery from the aorto-subclavian graft. The patient became completely symptom-free at clinical evaluation one year following surgery.

### Case 2

A 49-year-old female smoker presented with a two-year history of intermittent diplopia, dizziness and ataxia. On physical examination there was a diminished left radial pulse and a bruit was audible in the left supraclavicular region. Contrast-enhanced spiral volumetric CT images showed patchy calcification at the origin and along the course of all the great vessels. The first 10 mm segment of the left subclavian was completely occluded with retrograde filling of the left subclavian and vertebral arteries. She underwent the same procedure as Case 1 and made an uneventful recovery. Post-operative CT showed good antegrade flow through the graft to distal left subclavian artery. The patient remained symptom-free at follow up review.

### Case 3

**Figure 2:**
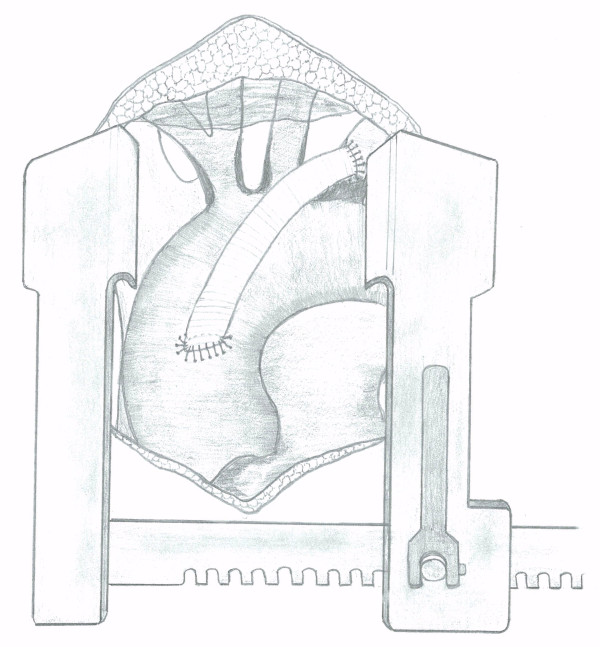
**Aorto-innominate Bypass Graft**.

A 68-year-old female presented with a 3-year history of progressively worsening angina. She then developed intermittent diplopia and subsequently complained of exertional right arm pain. Angiography showed triple vessel coronary artery disease and an occluded right innominate artery. Doppler ultrasound showed intermittent flow reversal in the right common carotid artery and retrograde flow in the right vertebral artery (subclavian and carotid steal). At operation, a standard median sternotomy was performed with a small extension of the incision into the neck. The innominate artery was clamped with a single partial occlusion clamp distal to the lesion and a 5 mm Goretex graft sutured to it under full heparinization (Fig 2). This was followed by standard triple coronary artery bypass grafting. The aortic cross clamp was removed and the innominate graft was attached to the aorta in a similar fashion to the proximal coronary anastomosis.

**Figure 3:**
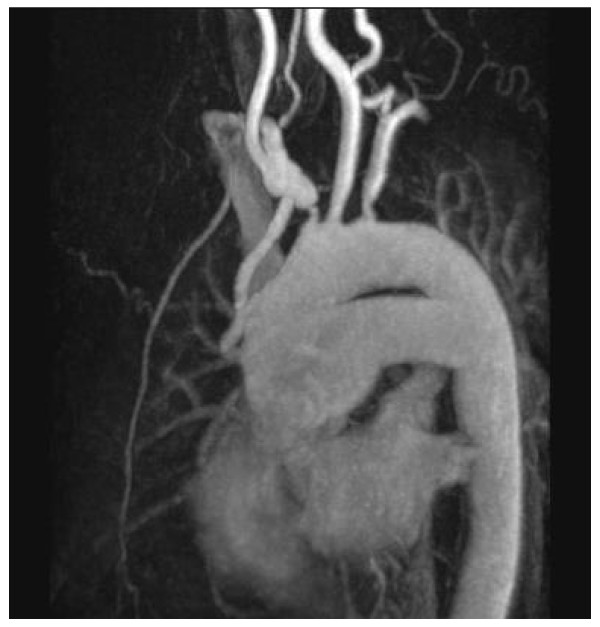
**Grafted Right Brachiocephalic Stenosis**.

Postoperative magnetic resonance imaging showed a patent aorto-innominate bypass with good antegrade flow in the right carotid and subclavian arteries (Fig 3). The patient had an uneventful recovery with complete resolution of all symptoms (angina, diplopia and exertional arm pain) on follow-up.

## Discussion

Subclinical aortic atherosclerosis may start as early as the second decade of life [2] and the commonest disease in the aorta and the SATs is atherosclerotic in causation. Lesions develop principally in high shear stress regions, which is in the zone of flow separation and is associated with whirlpools that form near the lateral wall of bifurcations. Plaques are relatively uncommon in ascending aorta but more common in arch and descending thoracic aorta [3]. Hypertension, diabetes mellitus, cigarette smoking, dyslipideamia and genetic preponderance are common risk factors for developing atherosclerosis and in combination have a greater than additive effect. The majority of patient with SAT stenosis are asymptomatic, but some patients may present with symptoms of vertebrobasilar ischaemia including episodes of dizziness, diplopia, ataxia, vertigo, limb claudication, paraesthesia and steal syndrome. Physical examination may reveal diminished pulse and decreased blood pressure (> 20 mmHg reduction compared to the normal side) in the affected limb. The subclavian steal syndrome is one of the best recognised presentations of SAT stenosis. It is more common on the left side, perhaps due to the acute angle at the origin of the left subclavian artery which may result in accelerated atherosclerosis from increased turbulence. SAT stenosis can be diagnosed by digital subtraction angiography, duplex scanning, contrast enhanced spiral CT, magnetic resonance imaging and arch aortography.

The concept of extra-anatomic bypass was first introduced in 1952 by Freeman and Leeds [4], when they used superficial femoral artery to carry blood from one femoral artery to other, and this procedure has now become a widely used and accepted method of revascularisation. The physiologic basis of extra-anatomic bypass reveals that inflow in the donor artery is the key factor that determines the haemodynamic effects of extra-anatomic bypass. If the inflow in the donor artery is below a critical level of 60%, it may be insufficient to supply adequate blood flow simultaneously to both the distal segment of the donor artery and the bypass graft. Moreover, the capacity of the donor artery to provide increased blood flow on demand may be compromised because of atherosclerosis or iatrogenic stenosis at the site of anastomosis. To ensure a good result the donor artery should be free of disease and every precaution should be taken to avoid anastomotic stenosis [5]. The increased flow demand following the extra-anatomic bypass is met by increased flow in donor artery proximal to the anastomosis and the flow remains essentially unaffected by changes in the outflow and hypotension. The only factor that leads to the phenomenon of vascular steal is restriction or obstruction of inflow in donor artery.

Endovascular techniques are increasingly used in the treatment of occlusive SAT disease because they are less invasive, may be performed under local anaesthesia and are associated with shorter hospital stay. The vascular patency rates reported in different studies are variable and there are no randomised trials comparing endovascular and open surgical approaches. The innominate artery may present as a challenging SAT lesion for interventional endovascular therapists, due to its larger diameter, and short length between its origin and its bifurcation and between the bifurcation and the take-off of vertebral artery [6]. In addition it is sometimes difficult to negotiate a very tight stenosis or occluded lesion through an endovascular approach and the long term benefits of these therapies are uncertain. Modarai et al. [7] reported a better patency and lower complication rate related to extra-anatomic bypass for SAT disease as compared to percutaneous endovascular intervention. In this series of 76 patients, with a mean follow up of 5 years, the extra-anatomic graft patency was 97% with no complications against 82% patency for the endovascular intervention with angioplasty with a rate of complication of 11%.

In the past, atherosclerotic SAT stenosis was treated with anatomic bypass between aortic arch and innominate, carotid and subclavian arteries. Graft patency was good but perioperative mortality and stroke rates were high [8]. This led to the introduction of safer extra-anatomic approaches. The most commonly used open procedures for SAT stenosis involve a cervical approach, which is ideally suited for single trunk disease that involves either subclavian or common carotid arteries [1]. The procedure may include endarterectomy, bypass grafting from ipsilateral carotid artery or the subcutaneous crossover axillo-axillary bypass and transposition of the subclavian to carotid artery. Subclavian transposition has the benefit of a single anastomosis and eliminates the potential thrombotic and infection risks associated with the use of prosthetic grafts or saphenous veins [9]^.^

In comparison to the cervical approach, the transthoracic approach may carry a relatively higher morbidity but the results may be more durable in atherosclerotic disease involving SATs [1].

Transthoracic direct aorto-subclavian and aorto-innominate bypass have advantages in selected patients, including those with atherosclerosis which spares the ascending aorta, those isolated supra aortic trunks stenosis involving long vessel segments and those with disease affecting potential donor arteries such as the ipsilateral or contralateral carotid. Thus aorto-SAT bypass can be used safely in patients with concomitant carotid disease with reduced cerebral risks, avoids endarterectomy and its attendant thrombotic risks and provides a more physiological blood flow pattern than axillo-axillary or carotid-subclavian bypass.

Manubriotomy is a small, cosmetically acceptable incision which is well tolerated and less painful than standard median sternotomy. This approach gives excellent access to ascending aorta, arch and proximal supra aortic trunk. Manubriotomy, being a less invasive technique, can be used safely in patients with reduced cardiopulmonary reserve, but caution should be taken in previous mediastinal surgery or irradiation.

In patients with concomitant coronary artery disease and vertebrobasilar ischaemia, both pathologies can be dealt with simultaneously using a standard median sternotomy, avoiding the risks associated with second operation. Proximal ascending aortic anastomosis is a commonly performed procedure in coronary artery surgery and the technique is well established. Atherosclerosis is a progressive disease and if one SAT stenosis is present, future stenosis may develop in the feeding vessel after carotid-subclavian bypass and axillo-axillary bypass. Using the ascending aorta eliminates this risk. Patients who have or will have coronary revascularisation using an internal mammary artery as a conduit present a special problem in subclavian and innominate stenosis, as functionally the vascular segment between the origin of subclavian artery and the coronary artery becomes part of coronary circulation [10]. In such patients, the myocardium may be dependent on subclavian flow and care should be taken to ensure that whatever procedure is carried out, satisfactory antegrade flow to the relevant subclavian artery can be assured for the long term or alternatives should be sought for the internal mammary graft to prevent a coronary-subclavian steal syndrome and myocardial ischaemia.

## Conclusion

In conclusion, SAT stenosis is an uncommon condition which may be treated safely and effectively using aorto-subclavian and aorto-innominate bypass in selected patients.

## Abbreviations

SAT: Supra Aortic Trunk.

## Competing interests

The authors declare that they have no competing interests.

## Consent

A written informed consent was obtained from the patient for the publication of this case report and accompanying images. A copy of the written consent is available for review by the editor-in-Chief of the journal.

## Authors' contributions

**AK **did the literature review, drafted the manuscript, drew the illustration and also cared for the patient in peri-operative period. **SAM **is the operating surgeon, helped in the critical appraisal and final approval of draft. Both authors have read and approved the final manuscript.
